# Testing for type 1 diabetes autoantibodies in gestational diabetes mellitus (GDM): is it clinically useful?

**DOI:** 10.1186/s12902-019-0373-4

**Published:** 2019-05-03

**Authors:** Michela Incani, Marco Giorgio Baroni, Efisio Cossu

**Affiliations:** 1 Endocrinology and Diabetes, Department of Medical Sciences and Public Health, University of Cagliari, Cagliari, Italy; 2 Endocrinology, Department of Experimental Medicine, Sapienza University of Rome, Policlinico Umberto I, 00161 Rome, Italy

## Abstract

Gestational Diabetes Mellitus (GDM) is the most common metabolic disorder in pregnancy, and it is associated with increased risk of morbidity in maternal-fetal outcomes. GDM is also associated with a higher risk to develop diabetes in the future. Diabetes-related autoantibodies (AABs) have been detected in a small percentage (usually less than 10%) of women with gestational diabetes. The prevalence in gestational diabetes of these autoimmune markers of type 1 diabetes (T1D) has been assessed in many studies, together with the risk of progression of AABs-positive GDM towards impaired glucose regulation (IFG or IGT) and overt diabetes after pregancy. The question whether it is necessary to test for T1D autoantibodies in all pregnancies with GDM is still debated. Here we examine the epidemiology of T1D autoantibodies in GDM, their clinical relevance in term of future risk of diabetes or impaired glucose regulation and in term of maternal-fetal outcomes, and discuss when it may be the most appropriate time to search for T1D autoantibodies in women with gestational diabetes.

## Background

### Epidemiology of T1D autoantibodies in GDM

Gestational Diabetes Mellitus (GDM) is the most common metabolic disorder in pregnancy, with prevalence between 2 and 17% depending on the genetic background of the studied population [1–3]. GDM is defined as carbohydrate intolerance diagnosed in the second or third trimester of pregnancy that was not clearly overt diabetes prior to gestation [4]. According to this definition, conditions leading to beta cell deficiency during pregnancy may reveal as GDM, triggered by the impairment in insulin action that physiologically appears during pregnancy, aimed at favouring fetal growth. Normally the β-cell pool adapts to physiological needs and increased functional demands [5]. However, if this state of insulin resistance (IR) is not compensated by an increase in beta-cell insulin secretion, it determines the appearance of GDM and provides a higher risk to develop type 2 diabetes (T2D) [6]. Epidemiological data shows that in a subgroup of women, estimated to be between 0 and 10% [7] of all GDM cases, carbohydrate intolerance is associated with the presence of autoimmunity against β-cells. In these women there is a higher risk of progression to type 1 diabetes (T1D) and/or Latent Autoimmune Diabetes of Adulthood (LADA) after pregnancy [2, 7–11]. In rare occasions, autoimmune diabetes makes it first appearance in pregnancy as diabetic ketoacidosis (DKA) [12, 13]. When DKA is encountered in pregnancy the possibility of unrecognized pre-existing diabetes (mostly autoimmune) should be strongly considered. Pregnancy itself is a condition that predisposes to ketoacidosis, for example through nausea and vomiting in the first trimester, or insulin-resistance and increased lipolysis in the second and third trimesters [14].

Islet-cell autoantibodies, the markers of beta-cell autoimmunity, are present in sera from women with GDM with variable frequency. The prevalence of diabetes-related autoimmunity in pregnancy is extremely variable depending on the type of the autoantibody under study, the method for detection, and the population under observation.

Many studies have assessed the prevalence of diabetes-related autoantibodies in women with GDM, searching for ICA (islet cell autoantibody), IAA (insulin autoantibody), GADA (glutamic acid decarboxylase autoantibody), IA-2A (tyrosine phosphatase-like islet antigen autoantibody) and, recently, ZnT8-A (Zinc trasporter 8 autoantibody).

In general, titres for all autoantibodies are lower in GDM patients than in cases of newly diagnosed T1D [6, 15–23] or in first-degree relatives of patients with T1D [24, 25]. These AABs’ titres are similar to those observed in LADA patients, and are considered indicative of a slow-developing autoimmune process in women with GDM that are positive for diabetes-related autoimmune markers [7, 26].

With regards to the prevalence of individual AABs, ICA studies showed a variable prevalence between 1 and 35% [7]. Nonetheless, because of technical (standardization) and methodological (test variability) issues, ICA are now less often measured [27, 28].

**Fig. 1:**
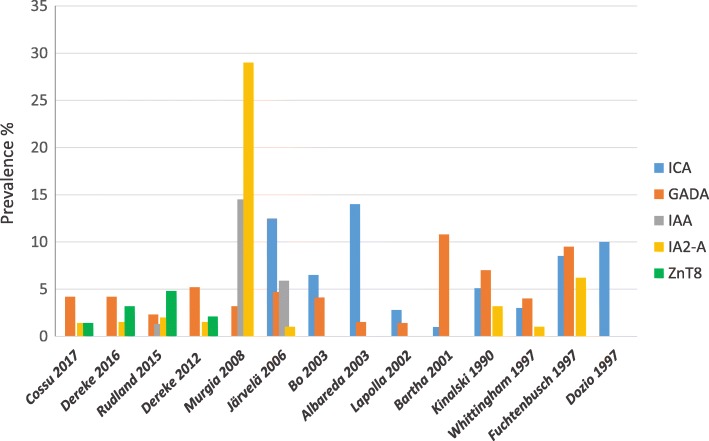
Autoantibodies prevalence in women with GDM. Prevalence of ICA, GADA, IA2-A and ZnT8-A in different studies

As for the other beta-cell autoantibodies, reports on the differences in autoantibodies frequencies and titres between GDM and control women have been conflicting, especially for GADA and IA2-A. The results of studies on GADA in GDM patients and controls vary widely; the overall frequencies of GADA range between 0 and 10.8% [7, 9, 19–22, 24, 29–43] (Fig. 1), with some studies showing higher frequency in GDM [9, 21, 22, 30, 39], and other no differences [19, 33–35, 43, 44]. Also GADA titres have been reported higher in GDM women in some studies [9, 21, 22, 30, 31], with others showing no difference [33–37]. As for the frequency of IA-2A positivity, this ranges from 0 to 6.2% (Fig. 1) [20, 21, 30, 38] with some papers [21, 30, 39] reporting a higher frequency in GDM patients than in normal controls (up to 26% in only one study [35], while others found no difference [9, 19]. Such contrasting results may be due to different genetic backgrounds within populations, since it is common knowledge that ethnicity plays an important role in determining beta-cell autoimmunity. Finally, a low frequency of IAA (Fig. 1) in GDM patients is reported in the literature [8, 15, 16, 19, 40], and only few studies found a different prevalence of IAA positivity in GDM patients than in the control women [9, 39], also confirming that IAA are more typical in younger ages [45].

The last beta-cell autoantibody to be discovered, ZnT8-A, was shown in a study by Rudland et al. to be the most represented in their GDM cohort, with a prevalence of 4.8%, also associating with higher basal blood glucose values compared to the other AABs [41]. In a more recent study Dereke and collaborators found that 6.8% of GDM women were autoantibody-positive, and 3.2% resulted ZnT8-A positive (Fig.1). Of the autoantibody-positive women with GDM who developed postpartum T1D, that were approximately 19%, all were positive for GADA. It seems therefore that ZnT8A does not have additional predictive values over GADA in the postpartum development of autoimmune diabetes [42].

Finally, very few studies have been conducted on populations with a high genetic risk for autoimmune diabetes, and they have shown a different prevalence of autoantibodies positivity in GDM between studies. For example, in a Finnish population, Jarvela et al. [9] observed a prevalence of autoimmunity of 16.7% in women with GDM vs. 2.8% in controls. Murgia et al. [39] reported in Sardinia a prevalence of 38.8% for at least one AAB vs 7.1% in control women; Cossu and co-workers [43] reported instead an overall prevalence of AABs of 6.4% in Sardinian women, a prevalence that was not different between GDM (5.6%) and control (8.3%) women (Fig. 1). This variability seems to be due to sample selection, screening criteria, different autoimmune measurement tools, all that could lead to conflicting results.

### Risk associated with AABs positivity to develop future T1D, LADA or impaired glucose regulation (IGR)

The development of an autoimmune or a non-autoimmune form of GDM depends on a pregnant woman’s genetic susceptibility. GDM may facilitate the identification of women at risk of developing diabetes later in life, and the presence of autoimmunity in GDM helps to estimate the risk of future type 1 or type 2 diabetes [46]. In particular, the risk of future T1D rises with increasing number of positive autoantibodies.

In this respect, it has been estimated that the risk of developing type 1diabetes two years post-partum is 17% in the presence of a single autoantibody, increases up to 61% in the presence of 2 autoantibodies, and to 84% when 3 autoantibodies are present [30]. In this study, among all antibodies tested, GADA had the highest accuracy in predicting autoimmune diabetes (sensitivity 63%) compared to ICA (48%) or IA2 (34%), but, overall, the presence of single GAD autoantibodies appeared to have limited predictive power, as described also for patients with LADA [26]. Löbner and collaborators reported that 96% of GDM women with at least one positive antibody (GADA or IA2-A) develop T1D within 8 years after delivery [47]. A Finnish population study [9] reports that 10% of women with a previous GDM develop type 1 diabetes within 6 years; the risk for type 1 diabetes correlates with an age under 30 years, with the need for insulin therapy during pregnancy, and with a positivity for at least 1 antibody. Another study reports an onset of T1D after 1 year of 50% in AABs-positive GDM women, and of 21% of IFG or IGT [10]. In a recent work from our group, women followed for 2 years after pregnancy had a 2.65 greater risk of having impaired glucose regulation when T1D-related autoantibodies where present, suggesting a continuing damage to beta-cells by the autoimmune process [43].

Overall, understanding the etiopathogenesis (i.e. autoimmune or non-autoimmune) of GDM is useful to evaluate the future risk of glycaemic alterations. The real question is “when it is useful to search for an autoimmune-diabetes related diagnosis?”

## When to search for T1D autoantibodies: is it useful during pregnancy?

The issue is whether autoimmune screening in women with GDM does offer clinical benefits, or, on the contrary, it may represent unnecessary health expenditure.

The overall prevalence of GDM has been constantly increasing through the years, reflecting the background prevalence of obesity and T2DM in the general population [48–51]. It is well known however that the autoimmune form of GDM represents a small proportion (0–10%) compared to all cases of GDM [2, 7]. A universal autoimmune screening in *all GDM women* may expose them to unjustified strain at a particular distressful time like pregnancy [52], also in consideration to the fact that GDM women with and without AABs have similar clinical outcomes (see below) [22, 41, 43, 53].

So far, data on the predictive power of individual or specific clinical features are discordant in literature; some authors, including ourselves, do not find dissimilarities in clinical features between women with and without autoimmune GDM [19, 43, 54, 55]. Other authors report that autoimmune GDM may be associated with a lower BMI, a more frequent need to receive insulin therapy and more severe clinical manifestations, such as higher blood glucose levels [30, 56]. However, data are inconclusive and the question is still open.

The screening for autoantibodies would probably be more indicated only when GDM is associated with the cluster of clinical features suggestive of a T1D-like form of GDM, for example when two or more parameters such as young age, low body mass index (BMI), early need for insulin therapy, presence of ketones, co-presence of other autoimmune condition (e.g. thyroiditis) are existing at the same time.

Also, several studies report no differences in maternal-fetal outcomes between women with or without autoimmunity [22, 41, 53], implying that hyperglycaemia per se (i.e. glucotoxicity for the fetus), whatever the cause, is the key determinant of these pregnancy outcomes [57]. This is an important point favouring not to measure autoantibodies during pregnancy in *all GDM women.* In a study by Lapolla et al. [40] pancreatic autoimmunity in GDM shows a low proportion, and it is shown that anti-GAD antibodies can, instead, appear after delivery. In fact, typical immunosuppression of the mother during pregnancy determines a reduction in the titres of some organ-specific antibodies. It is well known that, during pregnancy, the immune system shift towards a Th2-mediated immunomodulation, which is capable of suppressing autoimmunity and protecting from infections. Inversely, when in the post-partum period Th2-type cytokines decrease, worsening of Th1-mediated autoimmune disorders has been observed. The immune system is hence capable to influence autoimmunity in pregnancy, positively during the gestational period and negatively, even decades later, in the postpartum [58]. There is therefore the possibility to have false-negative cases if AABs are tested during pregnancy. Consequently, it has been proposed to carry out autoantibody re-valuation a few months after delivery [10], when it could be the time for a more useful autoimmune evaluation.

## When to search for T1D autoantibodies: is it useful after pregnancy?

As recommended by current guidelines, women with GDM should repeat an oral glucose tolerance test (OGTT) after 6–12 weeks from delivery; however, a significant percentage of these women do not repeat the OGTT [3, 59–62]. This observation highlights the need for more information to the patients on the long-term complications of GDM, in order to increase the chances for prevention of diabetes, metabolic syndrome and cardiovascular complications. The control of the metabolic state after childbirth in women with previous GDM is necessary for the future health of the woman [63]. It has to be strongly recommended by professionals who treat women at the time of pregnancy, also in order to assess the risk of future onset of diabetes.

Follow-up studies have show that overt diabetes appears usually from the first year after delivery onward. In the study from Nilsson et al. [64], for example, less than 20% of AABs-ve GDM women developed T1D within 1 year after pregnancy, with the vast majority developing T1D later. So, in the rare cases of T1D developing soon after delivery, following recommendation for an OGTT within 6–12 weeks from childbirth would prevent missing these cases.

In the context of the follow up, the evaluation of autoimmunity in women with a phenotype suggestive of an autoimmune form of diabetes may be indicated. As discussed above, the period after childbirth, compared to pregnancy, is a time more indicative of the real autoimmune status of women. Thus, based on results from others and ourselves, in the presence of persistent glucose impairment and in the absence of evident features of insulin-resistance, the determination of diabetes-related autoimmunity in the follow-up of GDM women should be recommended. Indeed, we observed a persistence of glucose abnormalities in AABs-positive women after almost 2 years of follow-up, which resulted in a 2.65 relative risk (RR) of glucose impairment in AABs-positive women [43]. Also Lundberg et al. [65] observed that the combination of OGTT and GAD autoantibodies post-partum identified women with impaired β–cell function, whom should be followed with special focus on the risk of developing autoimmune diabetes.

## Conclusions

From several epidemiological studies it appears that diabetes-related autoimmunity is not a main factor in the aetiology of GDM, accounting for less than 10% of all cases. GADA are the most common autoantibody compared to the other AABs, but autoantibodies show similar frequencies in GDM and NGT women [19, 31, 43, 44], suggesting that there is not a strong correlation between autoantibody-positivity and beta-cell impairment. In general [19, 54, 55], specific clinical features predicting which GDM women are at higher risk for autoimmune GDM have not been established, and it is therefore not possible to *exactly* define which patients to screen for autoantibodies during pregnancy.

Also, several studies report no differences in maternal-fetal outcomes between women with or without autoimmunity, suggesting that hyperglycaemia per se, whatever the cause, is the leading determinant of pregnancy outcomes.

Therefore, as in the Practice Points summarized in Table 1, unless a cluster of clinical features strongly suggestive of a T1D-like form of GDM (young age, low BMI, early insulin therapy, presence of ketones) is present, autoantibody screening may not be needed, and can be postponed to the follow-up of GDM women, when, for example, a persistent impaired glucose regulation is observed and may be indicative of an unremitting altered beta-cell function.

**Table 1:** Summary of practice points regarding autoimmune GDM in the clinical setting

PRACTICE POINTS	
• Islet cell autoimmunity is found in 0–10% of women with gestational diabetes. • GDM women with Islet-cell autoimmunity do not differ from women with non-autoimmune GDM with regards to treatment and pregnancy outcomes. • Autoantibodies screening in *all GDM women* is not recommended • Only if a cluster of clinical features strongly suggestive of a T1D-like form of GDM (two or more parameters amongst young age, low BMI, early insulin therapy, presence of ketones) is present, screening for autoantibody is recommended • The presence of islet autoimmunity during or after gestational diabetes predicts a higher risk to develop later impaired glucose regulation, type 1 diabetes or LADA. • Most women have normal glucose tolerance after delivery; if impaired glucose regulation persists, autoantibody screening is recommended. • Recommendations for the follow-up after GDM should be reinforced, given the higher risk to develop diabetes in the future	
